# Hirudiniasis in Cattle in Mpwapwa District, Dodoma Region of Tanzania

**DOI:** 10.1155/2020/3028345

**Published:** 2020-03-27

**Authors:** Misheck A. Mulilo, Joyce Siwila, Philbert B. Madoshi, Richard S. Silayo

**Affiliations:** ^1^The University of Zambia, School of Veterinary Medicine, P.O. Box 32379 Lusaka, Zambia; ^2^Livestock Training Agency, Mpwapwa Campus, P.O. Box 51, Mpwapwa, Dodoma, Tanzania; ^3^St. Francis University of Health and Allied Sciences, Department of Microbiology and Immunology, P.O. Box 175, Ifakara, Tanzania

## Abstract

Hirudiniasis is a condition caused by infestation by leeches. Despite the annoyance, clinical signs, and associated lesions caused by leeches in both humans and animals, the extent of the problem in Tanzania is not well elucidated. Individual animals and people become infested when they drink, bath, or walk through contaminated water sources. Leech infestations are more common in rural areas where water is scarce especially during the dry season. In this report, clinical cases of hirudiniasis in twenty-seven (27) cattle in Mpwapwa, Tanzania, are presented. The report further reviews the morphological features of leeches and creates awareness among the affected cattle farmers and the general public. A total of two hundred (200) live cattle were examined; out of these, 27 cattle had live leeches. There were, on average, 3–8 leeches per animal. Affected cattle had wounds on the gums, buccal mucosa, root of the tongue, and under the tongue with copious amounts of blood-stained frothy discharge. Further, the affected animals had respiratory distress and were rolling their tongues. The leeches were manually removed from all the affected cattle, with uneventful recovery. Laboratory examination revealed segmented, dorsal-ventral flattened, cylindrical parasites which had posterior and anterior suckers. The leech infestation was linked to drinking water from a pond which was suspected to be contaminated with “undescribed organisms” as indicated by the farmers. A follow-up was made and revealed no new cases and complete recovery of the affected animals. Hirudiniasis is one of the neglected diseases in humans and livestock and, therefore, requires integrated efforts especially in areas where water is scarce during the dry season. It is anticipated that this case report will stimulate interest and more studies in the subject matter to understand the extent of the problem and document the species and distribution of leeches in Tanzania.

## 1. Introduction

Hirudiniasis is a condition in which animals or people become infested by aquatic or terrestrial parasites called leeches. Leeches are blood-sucking ectoparasites with a worldwide distribution, affecting humans and wild and domesticated animals. They belong to the phylum Annelida and class Hirudinea [1]. They are dorsoventrally flattened with segmented bodies without an exoskeleton; however, their coela are not segmented as other annelids. They possess an anterior and posterior end, which are modified to form two prominent suckers, the anterior and posterior suckers, respectively. The posterior suckers aid in locomotion while the anterior suckers facilitate attachment to the host's body surface [2]. The anterior suckers in some species are modified to form well-developed three muscular jaws which are Y-shaped. The jaws are armed with a series of teeth-like structures which pierce the host's body surface during feeding [3–5].

Most species of leeches reside in water while a few species are telluric. The aquatic leeches are found in lakes, ponds, springs, slow-moving streams, marshes, and on moist vegetation in humid environments [6]. Common aquatic leeches include *Limnatis nilotica*, *Myxobdella africana*, *Dinobdella ferox*, *Hirudo medicinalis*, *Phytobdella catenifera*, and *Theromyzon tessulatum* [7] while *Haemadipsa zeylanica*, *Haemadipsa sylvestris*, and *Haemadipsa picta* are common telluric leeches [8].

Leeches have a direct life cycle where the eggs, which are laid in a cocoon, are internally fertilized and hatch to release young leeches. Depending on the species of leeches, cocoons may contain up to seven eggs and the former are deposited in muddy/dumpy soil or on rocks, plants, or other aquatic animals until the eggs hatch [9]. The cocoon acts as a protective capsule that provides a microenvironment necessary for embryonic development [10]. Immature leeches feed for a few days or several months (depending on the availability of hosts) before maturing into adult leeches [11, 12]. The leeches are hermaphrodites; however, they require another leech for fertilization to occur [9]. During fertilization, the male copulatory organ of one leech is inserted into the female copulatory organ of another [11]; hence, both leeches become fertilized concomitantly. Adult leeches can live for two to 27 years [9, 11].

Leeches have an exceptional feeding behaviour where they feed on copious blood from the host up to ten times their body weight within 20 to 40 minutes [12]. However, after taking a blood meal, they detach themselves from the animal and can stay without taking any more blood for 12 to 18 months [13]. Animals become infested when they drink leech-contaminated water while humans acquire the condition when they drink, bath, swim, or pass through leech-contaminated water. The leeches parasitize different parts of the body, where they invade internal body surfaces (hence, some are called internal leeches), while others localize on external surfaces (skin) hence named as external leeches [14]. Internal organs commonly parasitized include oral cavity, oesophagus, epiglottitis, nasopharynx, vagina, cervix, and ovaries [7].

In this study, twenty-seven (27) clinical cases of leech infestation in cattle in Mpwapwa, Tanzania, are presented and the gross morphological features of the isolated leeches are described. The information will create awareness among affected cattle farmers and the general public.

## 2. Case Report

Livestock Training Agency (LITA)-Mpwapwa campus in Tanzania conducts campaigns and sensitization on East Coast Fever immunization in the central zone of Tanzania. An arranged field visit was therefore made in May 2019 to Kisokwe village as part of the campaign. In total, 200 cattle were brought for immunization but it was noted that some appeared ill with peculiar clinical signs. The animals had acute respiratory distress and were drooling saliva which was frothy and blood tinged. A detailed history was then obtained from the farmers on the rearing systems from which it was noted that they practised extensive management system with a single communal watering pond-like stream. The farmers indicated that the stream in question was preferred as it was the only water source that was easily accessible during the dry season as other nearer sources dried up. The physical examination findings included reddish colouration of the oral mucosae, continuous chewing, rolling of the tongue, and bleeding from the mouth. In addition, one animal was severely emaciated and had intermittent bleeding from the nostrils and mouth. Furthermore, when some animals' mouths were held open, there was discomfort and the animals appeared to struggle to remove a “foreign body” from the mouth. The farmers speculated that the presenting clinical signs in the animals were due to drinking water from one of the streams.

**Figure 1:**
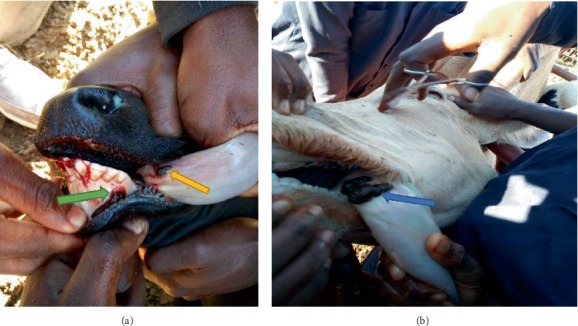
Parasites attached around the gums and under the base of the tongue (a) (green and yellow arrow, respectively); parasites on the torus linguae (b) (see blue arrow).

**Figure 2:**
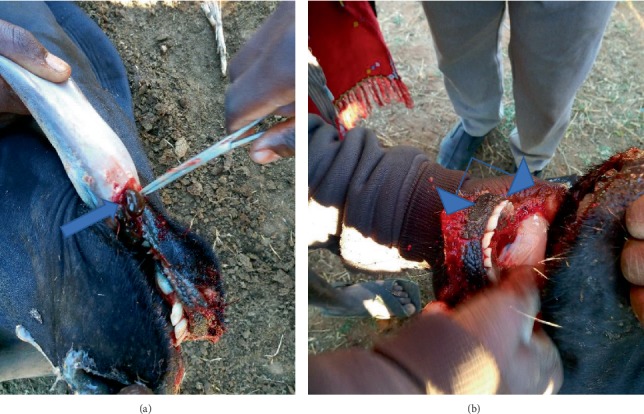
Parasites attached on the ventral side of the tongue (a); bloody froth in and around the mouth (b) (see the arrows*).*

Further clinical examination revealed the following: blood (frank blood) stained froth in the oral cavity, dark red flattened objects (average of 3-8 per affected animal). The organisms were longer than wider, attached themselves around the buccal cavity (surrounding the outer side of gums), under the tongue, and others were on the root of the tongue (Figures 1 and 2). Besides bleeding, open wounds were also seen on the gums and ventral aspect of the tongue.

**Figure 3:**
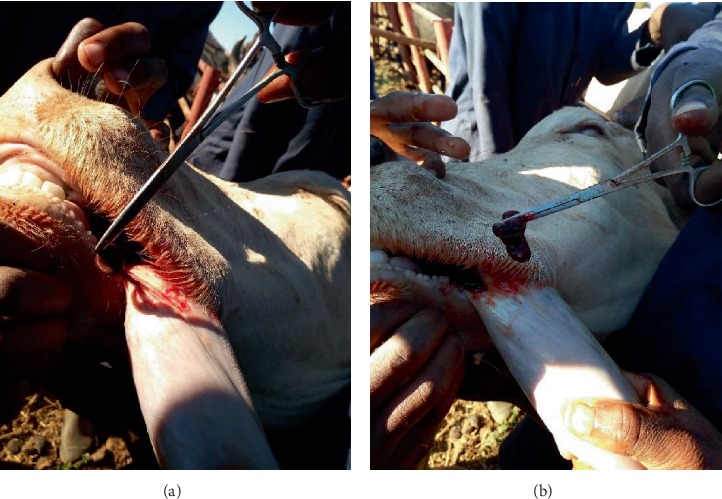
Leeches extracted from oral tissues with the aid of forceps.

The parasites were manually removed with the aid of forceps. Each parasite was grasped firmly at the third distal end from the anterior sucker and was gently pulled until detached from the animals' tissue (Figures 3(a) and 3(b)). The parasites were transferred to sample bottles containing 10% buffered formalin for further morphological identification in the laboratory.

Oozing of blood stopped after removal of the parasites and animals remained comfortable.

The collected parasites were transported to the laboratory for morphological identification. Laboratory examination was done using a stereomicroscope. The parasites appeared segmented, dorsal-ventral flattened, and cylindrical. The segments were of two forms, the large circular segments and small horizontal segments.

**Figure 4:**
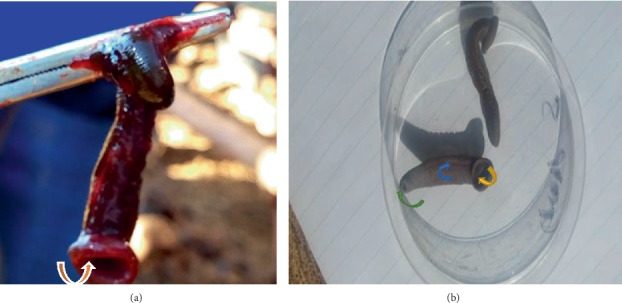
(a) Prominent anterior sucker seen on the flesh leech (see the arrow). (b) Ventral indentation (blue arrow), anterior sucker (yellow arrow), and posterior sucker (green arrow) on the leech.

**Figure 5:**
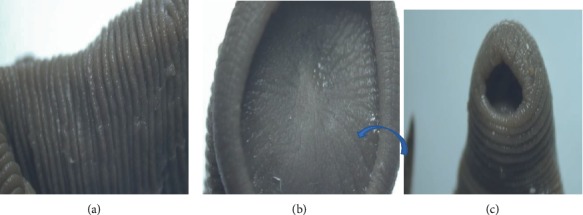
Indicating the external segmentation (a), anterior sucker (with line-line groves (see arrow)) (b) and posterior sucker (c) of the parasite under a stereomicroscope.

The circular segments marked indentation on the ventral side making two parallel continuous lines of the ventral side, running from the anterior to posterior end while small horizontal segments divided each circular segment (Figures 4(a) and 4(b)). The two extremities had suckers which were identified to be anterior and posterior. The anterior sucker was well marked with strong circular lips. On the inner surface of the lips were many grove-like lines which ran toward the centre (Figures 5(a)–5(c)). The anterior sucker had a circumference ranging between 15.7 mm and 40 mm while the posterior sucker was small with the circumference ranging between 2 mm and 6 mm. The parasites' body length ranged between 35 mm and 65 mm while their width ranged between 5 mm and 15 mm. It was also noted that the external segments did not correspond to an internal part of the body (Figure 4(b)). Based on these findings, the parasites were identified as leeches.

## 3. Discussion

This case report presents external features of leeches, leech infestation, and associated clinical signs in cattle in the central zone of Tanzania. Leeches have a worldwide distribution and are found in fresh and marine water and few of them on land. They have also been found in moist damp areas. They are blood-sucking, predatory, or scavenging ectoparasites both in humans and domestic and wild animals. Further, there are ubiquitous leeches that parasitize fish, frogs, and tortoise but can also attach to human hosts [15, 16].

In this study, leeches were recovered from the oral and buccal cavities of 27 cattle; common sites were around the gums, buccal mucosa, and under the tongue. Attachment on these sites has also reported in Iran and Ethiopia [17, 18], respectively. Other possible attachment sites are the ovaries, nostrils, and oesophagus [7].

In all cases, animals were dull, restless, and anorexic and were constantly rolling their tongues and showed vigorous chewing. Similar observations have been reported previously by Bahmani et al. [18]. Such health effects have a negative impact on animal performance especially draught power and dairy production. In the current case, animal production parameters were not assessed. However, there are some reports documenting leeches' contribution to reduction in productivity of livestock as a result of annoyances, discomfort, and lowered feed intake [19].

Despite the clinical signs and associated lesions in humans or animals, the extent of the problem in countries like Tanzania is not well elucidated. Based on the fact that water is scarce in most rural areas in developing countries, it is anticipated that the problem of leech infestation in Tanzania is either under reported or neglected due to lack of research on the subject. Nyamsingwa [20] reported zero prevalence of leech infestation in Ngorongoro in cattle, although livestock owners had knowledge of leech infestation both in animals and in humans. The infestation of leeches in this report was considerably high which could be attributed to animals drinking water from a pond contaminated by leeches in a study area which experiences water scarcity as was also described by Eguale et al. [21]. Silayo et al. [22] further described hirudiniasis to be one of the neglected zoonotic parasitic infestations because it affects humans and animals especially among rural dwellers.

It can be argued that infestation is more likely in the dry season because water becomes scarce in this season and is only found in a few spots. This is in line with other authors who reported that the amount of water can reduce the number of leeches in an area and that leeches can be a major constraint in livestock production during the dry season [23]. Further, Nyamsingwa [20] reported a low prevalence of leeches in water and animals during the rainy season in Ngorongoro.

Removal of the parasites is the only way to relieve the animals of the discomfort associated with leech infestation. A number of options are available and include drenching the animals with chloroform-water and manual extraction using forceps with/without local anaesthesia [21, 24]. In the current study, manual removal was done using forceps as described by other studies in Ethiopia [6], Libya [22], and Iran [18, 25]. However, animals should be prevented from drinking contaminated water as was done in the current case. Farmers were advised to water their animals from other sources which were not contaminated by leeches. However, the suggested sources were at a greater distance from their residences. On follow-up, it was established that the farmers obliged and there were no new cases.

Common chemicals such as copper sulfate, N-trityl-morpholine, and niclosamide have been used in water bodies to kill leeches, but they are toxic to other aquatic organisms, livestock, and human beings. However, the use of medicinal plants like endod (*Phytolacca dodecandra*) at a concentration of 20 g/m3 maintained for 6 hrs in water has been shown to give a significant reduction in the prevalence of the parasites in water bodies and is less toxic compared to chemicals [21, 26].

According to literature, there is no reported hirudiniasis case in humans from the central zone of Tanzania despite the semiarid nature of the zone. However, the lack of literature does not reflect the lack of the condition. Leech infestation in humans is associated with anaemia and reduction in the blood clotting prevention mechanisms [13, 14]. Anaemia in most cases affects women and children who are more likely to acquire the parasites due to their sociocultural behaviour in rural settings. Moreover, Kruger et al. [27] reported leeches to be the potential cause of severe anaemia in a 15-year child in Mbulu district in Tanzania, and this has been echoed by other studies elsewhere [3, 24, 28]. The public health importance of the parasites calls for more studies to investigate the extent of the problem in rural areas of Tanzania.

## 4. Conclusions

Based on these findings, it is clear that leeches are common parasites in the study area and probably associated with scarcity of water especially during the dry season. Therefore, hirudiniasis should be considered as a differential diagnosis in animals which present with oozing of blood from mouth, vigorous mastication, and rolling of the tongue. More studies should be done to determine the extent of the problem in Tanzania, and these should document the species and distribution of leeches bearing in mind that some leeches have beneficial clinical use.
